# Reticulophagy Reprograms the Endoplasmic Reticulum for SARS-CoV-2 Replication

**DOI:** 10.3389/fcell.2022.896618

**Published:** 2022-04-27

**Authors:** Xiaojuan Li, Ersheng Kuang

**Affiliations:** ^1^ College of Clinic Medicine, Hubei University of Chinese Medicine, Wuhan, China; ^2^ Institute of Human Virology, Zhongshan School of Medicine, Sun Yat-Sen University, Guangzhou, China; ^3^ Key Laboratory of Tropical Disease Control (Sun Yat-Sen University), Ministry of Education, Guangzhou, China

**Keywords:** reticulophagy, SARS-CoV-2, replication, endoplasmic reticulum, ER stress

## Introduction

The prevalence of severe acute respiratory syndrome coronavirus 2 (SARS-CoV-2) infection and the COVID-19 pandemic outbreak are causing overwhelming public health disasters worldwide. At the beginning of the pandemic outbreak, studies in cell cultures revealed that chloroquine and its derivatives, which are commonly used as autophagy inhibitors, significantly suppressed SARS-CoV-2 infection and replication (Liu et al., 2020; Wang et al., 2020). Although the lack of observed benefit in clinical COVID-19 therapies was confirmed through phase III clinical investigation (Borba et al., 2020; Tang et al., 2020), the importance of autophagy during SARS-CoV-2 infection and autophagy-related therapeutic strategies for developing COVID-19 treatment have been generally accepted.

Studies have revealed that SARS-CoV-2 infection hijacks autophagy machinery through different mechanisms (Zhang et al., 2021a; Hayn et al., 2021; Qu et al., 2021; Shang et al., 2021). Different viral proteins activate early autophagy or block late autophagy and autophagic flux and thus lead to the accumulation of autophagosomes, which play essential roles in viral replication and virion egress. Importantly, the mobility group box 1 (HMGB1) is a Beclin1-binding protein and acts as an autophagy effector (Tang et al., 2010), the viral protein ORF3a activates autophagy initiation through HMGB1- and UVRAG-mediated Beclin1 pathways (Qu et al., 2021; Zhang et al., 2022), while inhibiting autophagosome-lysosome fusion and autolysosome maturation by disrupting the VPS39 and HOPS complex (Zhang et al., 2021b; Miao et al., 2021). In summary, ORF3a selectively induces incomplete autophagy during SARS-CoV-2 infection. Interestingly, the homolog ORF3a in SARS-CoV-1 fails to induce autophagy (Qu et al., 2021; Zhang et al., 2022), indicating that this mechanism of autophagy induction specifically occurs during SARS-CoV-2 infection but is not common in other coronavirus infections such as SARS-CoV-1.

**FIGURE 1:**
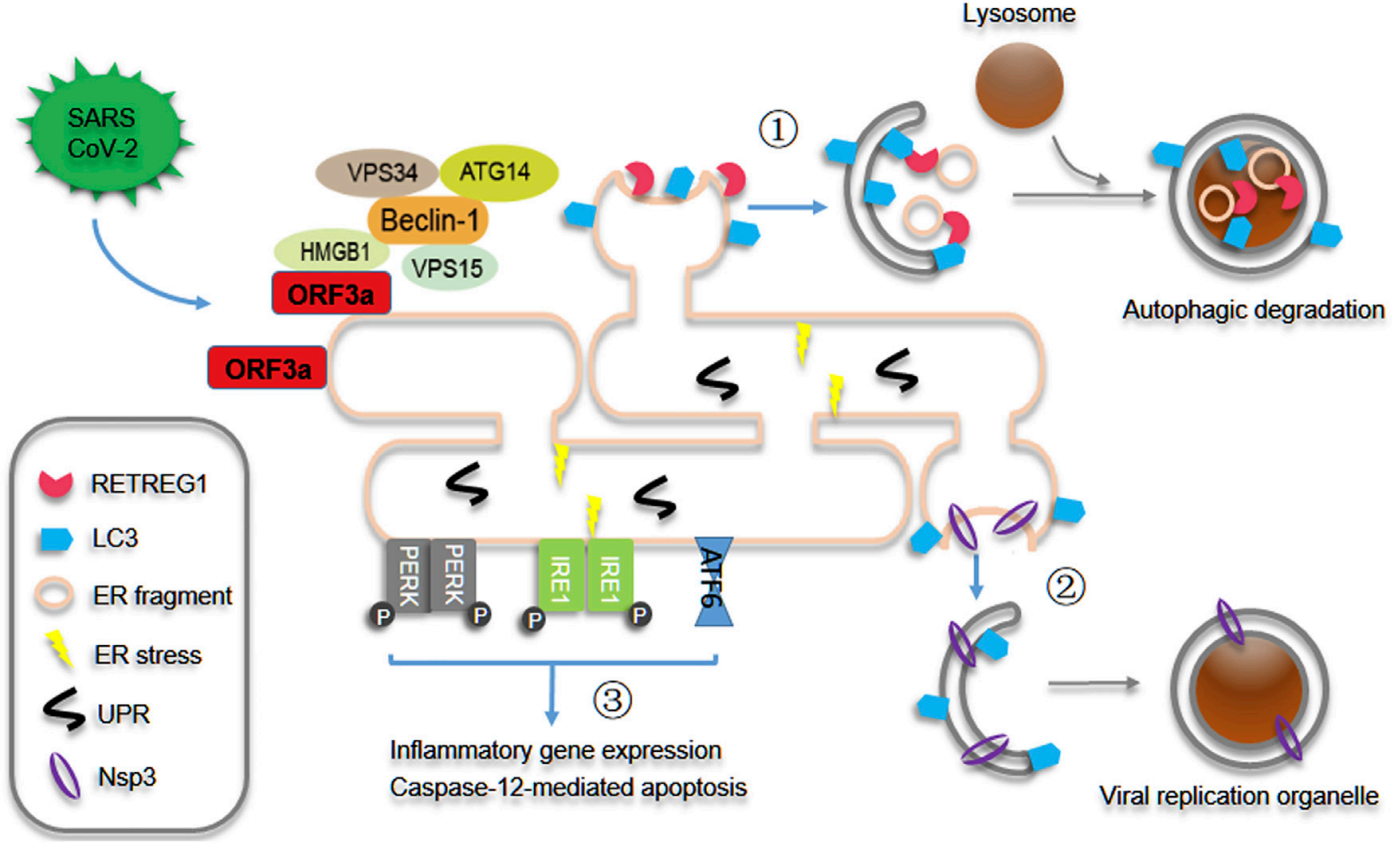
SARS-CoV-2 ORF3a induces reticulophagy and reprograms ER homeostasis. During SARS-CoV-2 infection and replication, ORF3a interacts with HMGB1 and recruits its translocation from nucleus to ER and then promotes HMGB1-Beclin1 association and RETREG1/FAM134B-mediated reticulophagy. As results ① ER integrated proteins and fragments are degraded and recycled through reticulophagy, and ② Nsp3-enriched ER regions are engulfed to form the double-membrane viral replication organelles probably through autophagy-like mechanism and then facilitates viral replication. Consequently ③ reticulophagy induces ER stress, and then triggers inflammatory gene expression and early caspase-12-mediated apoptotic phenotype.

As the critical site for cellular and viral protein synthesis inside cells, the endoplasmic reticulum (ER) plays the essential roles in SARS-CoV-2 infection and replication. Cellular protein synthesis is impaired by several viral proteins such as Nsp1 and Nsp14 through multiple strategies, and then efficient viral translation and evasion of innate defenses are enabled through inhibiting cellular translation (Schubert et al., 2020; Finkel et al., 2021; Hsu et al., 2021), and viral proteins rush into the ER compartment and support viral replication, virion assembly and transport. Our recent study showed that SARS-CoV-2 ORF3a localizes to the ER compartment, induces HMGB1 translocation from the nucleus and then recruits HMGB1 to the ER compartment, subsequently ORF3a promotes HMGB1-Beclin1 association and induces autophagy through Beclin1-dependent pathway. Therefore, ORF3a triggers reticulophagy regulator 1 (RETREG1)/FAM134B-mediated autophagy of the endoplasmic reticulum (reticulophagy) during SARS-CoV-2 infection (Figure 1) (Zhang et al., 2022). The degradation of ER membrane proteins is enhanced by ORF3a expression and SARS-CoV-2 infection, while the turnover of mitochondrial membrane proteins is not affected, indicating that ORF3a selectively triggers reticulophagy but not mitophagy during SARS-CoV-2 infection. Mitophagy is triggered by ORF10 and M overexpression, which are localized to mitochondria, to induce MAVS degradation (Li et al., 2022) and suppress antiviral immune responses (Hui et al., 2021) during SARS-CoV-2 infection. In contrast, galectin-8, one of cytosolic lectins, acts as a pattern and/or danger recognition receptor for intracellular pathogens and mediates selective autophagy against bacterial and viral infection (Thurston et al., 2012; Montespan et al., 2017), galectin-8 senses highly glycosylated viral proteins, such as SARS-CoV-2 spike, and then triggers antiviral xenophagy or virophagy, while SARS-CoV-2-encoded 3CLpro cleaves galectin-8 and the adaptor FYCO1 and thus disrupts xenophagy to evade antiviral autophagy (Pablos et al., 2021). These findings indicate that pro-viral autophagy is induced while antiviral autophagy is inhibited through different mechanisms during SARS-CoV-2 infection and replication. Because several viral proteins can also regulate autophagy through other mechanisms, such as ORF3a interacting with heme oxygenase 1 (HMOX1) and probably regulating autophagy under oxidative stress (Zhang et al., 2022), it is interesting to further investigate whether other kinds of autophagy are regulated by SARS-CoV-2 infection.

ER stress is induced by ORF3a expression and SARS-CoV-2 infection through HMGB1-dependent RETREG1 -mediated reticulophagy, subsequently triggering acute inflammatory responses and acquisition of an early ER apoptotic phenotype. These results indicate that ORF3a-induced reticulophagy promotes ER turnover and reprograms ER homeostasis during SARS-CoV-2 infection, probably to attenuate inappropriate ER responses to overloaded or unfolded viral proteins, recover from ER stress or repair ER damage. Many viral proteins primarily induce the unfolded protein response (UPR) and ER stress and secondarily trigger autophagy (Sharma et al., 2017; Yuan et al., 2017; Lee et al., 2018); however, ORF3a and SARS-CoV-2 infection induce reticulophagy in a different way. Our studies have excluded the possibility that ORF3a induces reticulophagy in a UPR- or ER stress-dependent manner. The truncated ORF3a construct that fails to interact with HMGB1 fails to induce reticulophagy and ER stress, and ORF3a interaction with HMGB1 alone or ORF3a localization to the ER membrane alone was not sufficient for autophagy induction. On the other hand, FAM134B-mediated reticulophagy is required for ORF3a-induced ER stress, while the activation of ER stress, at least the activating transcription factor 4 (ATF4)-CCAAT/enhancer-binding protein homologous protein (CHOP) arm, is not essential for the autophagy induced by ORF3a overexpression. These results confirmed that ORF3a primarily induced reticulophagy through the HMGB1-ORF3a interaction and ER membrane localization and then secondarily induced ER stress through reticulophagy (Zhang et al., 2022).

Autophagy may exert either pro-viral or antiviral effects during different viral infection. Studies have revealed that autophagy induction is essential for the infection and replication of SARS-CoV-2 and coronaviruses (Maier and Britton, 2012; Carmona-Gutierrez et al., 2020; Miller et al., 2020). HMGB1 depletion or RETREG1 depletion significantly suppresses the level of SARS-CoV-2 gene expression, indicating that HMGB1-mediated reticulophagy is essential for SARS-CoV-2 infection or replication. Why does the replication of SARS-CoV-2 and coronaviruses require autophagy and reticulophagy? First, coronaviruses replicate in double-membrane vacuoles named viral replication organelles (RO) (Wolff et al., 2020a), which are likely autophagy-related vacuoles or are generated through autophagy-like machinery. As reported by several groups, ORF3a expression and SARS-CoV-2 infection trigger incomplete autophagy (Zhang et al., 2021b; Miao et al., 2021; Qu et al., 2021; Su et al., 2021) and then cause the accumulation of autophagosomes and immature autolysosomes, which may promote RO formation or provide membrane sources for the double-membrane vacuoles. Second, several viral proteins are localized in the ER compartment (Santerre et al., 2021) and remodel ER morphology to facilitate viral assembly and transport. Third, reticulophagy and ER stress may exert pro-viral responses to promote viral replication and assembly (Zhang et al., 2022). Finally, recent studies have shown that SARS-CoV-2 ORF3a blocks autophagosome-lysosome fusion and promotes viral egress through lysosomal exocytosis (Chen et al., 2021; Miao et al., 2021), emphasizing that the subversion of autophagic or lysosomal vacuole translocation is important for virion transport and release. These findings indicate that autophagy and autophagy-related vacuoles play essential roles in the replication of SARS-CoV-2 and coronaviruses through multiple mechanisms.

Interestingly, the induction of reticulophagy by SARS-CoV-2 infection is opposite to that of flaviviruses (e.g., Zika). Zika virus NS2B3 cleaves RETREG1/FAM134B to suppress reticulophagy and remodel ER morphology and then establishes ER-localized viral replication compartments (Lennemann and Coyne, 2017; Evans et al., 2020), which are mainly derived from the ER through the formation of intermediate single-membrane structures (Wolff et al., 2020b). In contrast, SARS-CoV-2 directly induced the formation of double-membrane RO structures derived from the ER, probably through an autophagy-like mechanism, and then replicated in this kind of autophagosome-like vacuole (Knoops et al., 2008; Wolff et al., 2020a). Therefore, reticulophagy and ER turnover exhibit the opposite effects in the replication of SARS-CoV-2 and Zika virus. However, it remains unknown whether reticulophagy is commonly induced by infection with coronaviruses for the formation of viral replication organelles.

In summary, SARS-CoV-2 infection induces RETREG1-mediated reticulophagy through ORF3a interacting and recruiting HMGB1-Beclin1 complexes to the ER compartment and then reprograms ER homeostasis, probably through both ER morphology and responses to ER stress, to facilitate viral replication and trigger the proinflammatory responses. These findings represent novel insights into the induction and function of reticulophagy in SARS-CoV-2 infection and provide important targets for developing autophagy-related COVID-19 treatment.
